# The impact of anxiety on the cognitive function of informal Parkinson’s disease caregiver: Evidence from task-based and resting-state fNIRS

**DOI:** 10.3389/fpsyt.2022.960953

**Published:** 2022-09-08

**Authors:** Hai-Yang Wang, Lu Ren, Tao Li, Lanlan Pu, Xiaofeng Huang, Song Wang, Chunli Song, Zhanhua Liang

**Affiliations:** Department of Neurology, The First Affiliated Hospital of Dalian Medical University, Dalian, China

**Keywords:** caregiver, Parkinson’s disease, anxiety, cognitive function, fNIRS

## Abstract

Informal Parkinson’s disease (PD) caregivers are considered to experience high levels of caregiver burden, negatively affecting the health of caregivers. However, few studies explored the relationship between anxiety in caregiver burden and cognitive function in informal PD caregivers. Although, no study has even investigated the neural mechanisms underlying this connection. This study aimed to conduct comprehensive cognitive and clinical assessments and evaluate brain activity from task-based state and resting-state using functional near-infrared spectroscopy (fNIRS). A total of ten informal PD caregivers and 15 matched, healthy, non-caregivers were recruited. Comprehensive cognitive and clinical assessments were conducted to evaluate five cognitive domains and mental states. Neural activity induced by verbal fluency task (VFT) and brain connectivity during resting state were monitored, and their correlations with the neuropsychological and clinical tests were explored. Our results showed that compared to non-caregiver, an informal PD caregiver exhibited no difference in most cognitive domains of function but performed better in attentional function, along with higher levels of anxiety. Decreased activation over prefrontal regions during VFT and hypo-connectivity within the frontoparietal network (FPN) and between default mode network (DMN) and FPN in the resting state were confirmed in this study as a result of the negative effects of anxiety on the brain. Furthermore, Spearman’s correlation found that neural activity in FPN during task-based state and resting state was negatively correlated with the severity of anxiety. These findings indicate that despite normal or even better cognitive function, informal PD caregivers have impaired brain function, and this deficit in neural activity was related to anxiety.

## Introduction

Parkinson’s disease (PD) is a chronic, progressive, and the second most common neurodegenerative condition in the world, affecting the lives of both the individuals and their families (1, 2). Patients with PD suffer from various motor and non-motor symptoms, leading to increased disability, high-cost medical care, and poor quality of life (3). Patients, therefore, need growing support, which is often provided by informal caregivers, generally unpaid spouses, children, or other relatives (4). Informal caregivers are likely to experience negative caregiving outcomes, such as sleep disturbance, chronic stress, and reduced life satisfaction associated with their caregiving obligations (5).

While many studies focused on the influence of caregiver burden across a variety of health outcomes (4, 5), few kinds of research have explored the impact of anxiety on cognitive function in informal PD caregivers (6, 7). Sound cognitive function is essential to daily living, including work, social interaction, and independence. In addition, poor executive function of the caregiver could adversely influence the lives of both the caregiver and the care recipient. Even minor executive function problems could impact the caregiver’s ability to provide appropriate care, which in turn impacts their overall quality of life (8). Notably, research showed that emotional illness is more associated with informal PD caregivers than the general population (9). Despite functioning relatively well physically, informal PD caregivers experience high levels of caregiver burden, such as anxiety (9, 10), which could lead to cognitive decline (11). Thus, it is significant to explore the impact of anxiety on cognitive function, especially the executive function, in informal PD caregivers.

To date, only a few studies, using an abbreviated assessment model of the neuropsychological scale, found that informal PD caregivers perform normally in cognitive function (6, 7). In-depth and comprehensive neuropsychological investigations may contribute to further characterizing the cognitive functioning of informal PD caregivers. Contrarily, the cognitive function of non-PD caregivers has been studied more. Some studies found that caregivers performed worse on assessments of executive function and working memory, the key domain of cognitive function, compared with the non-caregivers (12, 13). However, other studies pointed to different and opposite findings, suggesting that caregiver cognition is not different and even associated with better cognitive functioning compared to non-caregivers (14, 15). The reasons for these inconsistent results may be associated with age, education, caregivers’ health status, and sample size (14).

While significant information about altered cognitive abilities in caregivers has been uncovered by prior investigations, the simple application of standardized neuropsychological assessments may limit our ability to understand the complexity of the underlying impairments, as the impact of cognitive function on neural cognitive processing is more complex than behavioral performance (16). Recent developments in neuroimaging techniques, particularly functional brain imaging, have expanded our understanding of the underlying neural basis of cognitive function (16, 17). Using task-based functional magnetic resonance imaging (fMRI), prefrontal regions associated with executive function were identified, including the dorsolateral prefrontal cortex (DLPFC) and the ventral lateral prefrontal cortex (VLPFC), the orbitofrontal cortex (OFC), and the sensorimotor areas (SMA) (18, 19). Resting-state functional connectivity (RSFC) in networks, such as the FPN (also known as the central executive network) involving the frontal lobes and the inferior parietal lobule (IPL) and the DMN involving the medial prefrontal cortex (mPFC), OFC, posterior cingulate cortex, and temporal lobe (TL) (20), are related to the cognitive function, including execution function (21–23). These observations suggest that cognitive activities recruit the frontoparietal brain regions for cognitive control, a brain mechanism occurring not only in the task state but also in the resting state.

However, no studies using functional imaging techniques have been performed to explore cognitive function in informal PD caregivers. Functional near-infrared spectroscopy (fNIRS) is an optical imaging technique used to non-invasively measure changes in the cerebral hemoglobin concentration elicited by neural activities (24) based on neurovascular coupling similar to fMRI. A task-based activity refers to neural pathways of the brain to specific stimuli, such as executive function and emotion (25). A resting-state functional activity indicates spontaneous fluctuations in regional brain activity without stimulation or goal-directed activity (26). fNIRS has become a well-established technology for cognitive neuroscience research (24). Task-based and resting-state fNIRS have been widely utilized to explore brain cognitive functions (27, 28).

In the present study, we made the first attempt to investigate the neural response of VFT associated with executive function and functional connectivity in resting-state networks in informal PD caregivers by fNIRS. Based on the subject’s knowledge background, a comprehensive evaluation of the cognitive function, including executive function, language, attention, memory, and visuospatial function, was conducted. Past research suggests that chronic stress, such as anxiety, could impair executive function, leading to diminished prefrontal activation and decreased functional connectivity in the resting state (29). Based on the studies described above, we hypothesized that, under VFT, the prefrontal regions, involving the DLPFC, the VLPFC, the OFC, and the SMA are not well activated in PD caregivers when compared with the general population. Moreover, reduced RSFC was found in FPN and DMN in informal PD caregivers.

## Materials and methods

### Participants

A total of ten informal caregivers of individuals with PD and 15 healthy individuals (non-caregivers) were recruited from the First Affiliated Hospital of the Dalian Medical University and the surrounding community. Given that this is the first fNIRS study examining cognitive function in informal PD caregivers, the required sample size could not be defined from *a priori* power analyses. Thus, we determined the required size of the sample for the PD informal caregiver group as 10 referencing three recent fNIRS studies exploring cognition (30, 31). Informal PD caregivers were recruited in seven cases when they brought patients for follow-up and in three cases when they accompanied patients in the inpatient ward. The average time the caregivers took care of patients with PD was 3.1 ± 1.4 years. We defined informal PD caregivers as primary caregivers of patients with PD including spouses and relatives and living independently in the community to help dependent patients with PD with some activities of daily living, while non-caregivers were defined as individuals with no caregiving activities. All primary caregivers are identified through self-reporting and are not required to live with the patient with PD (9 caregivers live with the patient and 1 does not live with the patient). All participants were required to have normal or corrected normal hearing and visual acuity. Participants were excluded if they had any medical or serious mental illness that could significantly impact their cognitive function. Since the time of day could affect cognitive abilities, the timing of the fNIRS examination is similar for informal PD caregivers and non-caregivers.

### Neuropsychological assessment

All individuals received a comprehensive neuropsy-chological evaluation, involving the Mini-Mental State Examination (MMSE) for global cognition and standardized measures assessing five cognitive domains: (1) executive function using the Clock Drawing Test (32) and the Semantic Fluency Test (animals) (33); (2) language using the 30-item Boston Naming Test (34) and the Similarities Test (35); (3) attention/working memory using the Symbol Digit Test and the Digit Span Test (35); (4) episodic memory using logical memory (36) and the Auditory Verbal Learning Test (37); and (5) visuospatial function using the Clock copying (Royall’s CLOX 2) (38) and the Block Design Test (35).

All participants completed the Hamilton Anxiety Rating Scale (HAMA) (39) and the Hamilton Depression Rating Scale (HAMD) (40). We performed the fNIRS measure immediately after completing the neuropsychological assessment for all subjects. The delay between the fNIRS measure and the neuropsychological assessment was 0 to 1 h. All of the procedures were conducted with adequate understanding and after obtaining written consent of the participants following the Declaration of Helsinki. Ethical approval was obtained from the Ethical Committee of the First Affiliated Hospital of Dalian Medical University.

### Task description

A Chinese version of VFT (a letter version) was used in our study. Detailed descriptions of the experimental tasks are available in another research (41). Briefly, the task involves 3 phases, including a 30s pre-task period, a 70s post-task period, and a 60s task period, consisting of three 20-s blocks. The participants were required to repeatedly count the numbers from 1 to 5 verbally during the pre- and post-task periods. Three Chinese characters (‘江,’ ‘日’ and ‘家,’ indicating ‘river,’ ‘day,’ and ‘home,’ respectively) were displayed to the subjects as sounds in each 20-s block, and the subjects were required to produce as many words starting with the same Chinese character as possible. The total number of correct words spoken by the participants was recorded as the VFT behavioral data.

### Resting-state paradigm

In the resting state, participants sat in a comfortable chair in a quiet room. They were asked to sit with their eyes closed, without falling asleep, and not think about anything as far as possible, and the collection of the NIRS data continued for 7 min for each participant.

### fNIRS measurement

**FIGURE 1:**
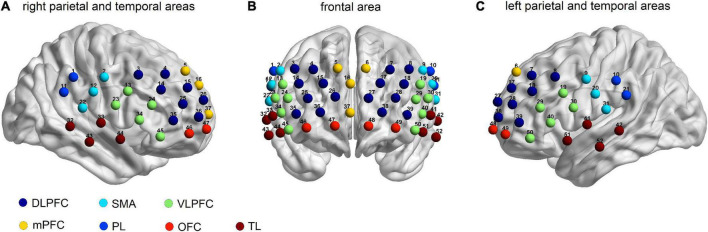
Locations of the 52-channels for the functional near-infrared spectroscopy (fNIRS). Estimated cortical areas corresponding to each channel using the virtual registration method in **(A)** the right parietal and temporal areas, **(B)** the frontal area, and **(C)** the left parietal and the temporal areas.

A 52-channel NIRS optical topical topography system (ETG-4000, Hitachi Medical Co., Japan) with a temporal resolution of 10 Hz was employed. This system measured the relative changes in oxygenated hemoglobin (oxy-Hb) and deoxy-hemoglobin (deoxy-Hb) using 2 wavelengths (695 nm and 830 nm) based on the improved Beer-Lambert law (42). A total of 17 light emitters and 16 light detectors arranged in a 3 × 11 array with a distance of 3.0 cm between them were included in this system. Optodes were deposited on the forehead and the scalp, and the lowest optode was placed along the T4-Fpz-T3 line according to the international 10-20 system used in electroencephalography. To get the correspondence between the measured channels and the locations on the cerebral cortex, a virtual registration method was employed based on the international 10-20 system (Figure 1; 43, 44). The exact coordinates of the 52 fNIRS channels are described in Takizawa et al. (45). The regions of measurement covered the bilateral DLPFC [Channels (Ch) 3, 4, 7, 8, 14, 15, 17, 18, 25, 26, 27, 28, 35, 36, 38, and 39], the VLPFC (Ch 13, 19, 23, 24, 29, 30, 34, 40, 45, and 50), the mPFC (Ch 5, 6, 16, and 37), the OFC (Ch 46, 47, 48, and 49), the TL (Ch 32, 33, 41, 42, 43, 44, 51, and 52), the sensorimotor area (Ch 2, 9, 12, 20, 22, and 31), and the IPL (Ch 1, 10, 11, and 21), consistent with the previous fNIRS study (46). Since the lateral OFC is usually regarded as the VLPFC, we define CH 45 and 50 as the VLPFC (47).

### fNIRS processing and statistical analysis

For task-based data, analysis of raw fNIRS data was conducted using the NIRS-KIT software package (48) based on the MATLAB environment (The Mathworks, United States). We focused on oxy-Hb (as opposed to deoxy-Hb), in which the levels more directly reflect cognitive activation and correlate more strongly with the fMRI blood oxygenation level-dependent signals (49). The optical density data were converted to changes in oxy-Hb concentration using a modified Beer-Lambert law (42). Then, the data were corrected by using the first-order detrending method, eliminating the linear baseline drift caused by long-term physiological changes or unstable devices. Head motion was corrected through a temporal derivative distribution repair (TDDR) algorithm (50). Butterworth band-pass filter (0.006-0.2 Hz) was applied to eliminate unrelated low- and high-frequency components. A General Linear Model (GLM) was used to evaluate the change in the oxy-Hb concentration for each channel on each individual. The task-period conditions were convolved with the standard typical hemodynamic response function (HRF) in the GLM model to form the corresponding regressors, and 10 s at the end of the pre-task period and 5 s after the 50-s post-task period were set as baseline (41). The task-related β-value was determined as the β-value of the generated words minus the β-value of the baseline. Then, the individual VFT-induced activation (β-value) was calculated for every channel. Intra- and inter-group comparisons of β values between the two groups were conducted by a one-sample test and a two-sample *t*-test (the region of interest was FPN), respectively.

For the resting-state data, RSFC analysis was performed using the NIRS-KIT (48) in a MATLAB environment (The Mathworks, United States). The first 10 s and the last 10 s of data were dropped to get a stable signal. Detrending and motion correction in preprocessing were the same as the above task-state processing. Butterworth band-pass filter (0.01-0.08 Hz) was applied to eliminate unrelated components. Then, the functional matrix was obtained by calculating the Pearson correlation coefficient (*r*-value) of the time series for each channel. Fisher’s r-z transformation method was used to convert the obtained r-values to z-scores to create a 52 × 52 correlation matrix. For each group, a one-sample *t*-test was used to generate a group-specific RSFC map. An independent samples *t*-test was conducted to evaluate the difference in FC between the PD-caregiver and healthy control groups.

Since this is an exploratory study, the significance threshold was determined to be 5% without false discovery rate (FDR) correction. Meanwhile, the significance of the difference after adjusting for FDR was also checked (51).

The χ2 test was used for the comparison of categorical variables. Clinical variables conforming to a normal distribution were compared using the *t*-test, while non-normally distributed clinical variables were compared by the Mann-Whitney U test.

For correlation analysis, the brain region where significant changes between two groups occur was selected as the region of interest. First, we tested the correlations between the change in the oxy-Hb level (β-value) during VFT and various clinical variables, including the HAMA, the HAMD, the MMSE, VFT performance, and executive function tests (Clock Drawing Test and Semantic Fluency Test) in the PD-caregiver group. Second, the correlations between RSFC and various clinical variables, including the HAMA, the HAMD, the MMSE, VFT performance, and 10 scales for cognitive domain assessment were performed in the PD-caregiver group. Pearson’s correlation coefficient was used and statistical significance was assumed when *pp*-value was < 0.05. Statistical analyses were conducted utilizing SPSS (version 26; IBM Corporation, Armonk, NY, United States) and the NIRS-KIT software package (48).

## Results

### Demographic and clinical data and behavioral performance

The demographic and clinical variables are summarized in Table 1. No significant differences were observed between the two groups in terms of age, gender, BMI, and education level. The mean HAMA score for PD caregivers was 8.7 ± 3.4, which was significantly higher compared to non-caregivers (*p* = 0.006), suggesting a state of anxiety. The HAMD scores were not significantly different between the two groups. Although no differences in the MMSE scores were observed, differences appeared in the comprehensive cognitive domain screen, exhibiting better performance in the Symbol Digit Test (*p* = 0.032), the Digit Span Test (total) (*p* = 0.058, marginally significant), and Backward digit span (*p* = 0.035) in PD caregiver compared to non-caregiver. There was no significant difference in the behavioral performance of VFT between the two groups.

**TABLE 1:** Demographic characteristics of informal PD caregiver and non-caregiver.

	Informal PD Caregiver *n* = 10	Non-caregiver *n* = 15	Group difference *p*-value
Age (year)	55.4 ± 10.5	61.1 ± 7.1	ns
Gender (Men/Women)	3/7	8/7	ns*^a^*
Years of education (years)	11.1 ± 3.0	10.3 ± 2.9	ns
BMI	24.8 ± 3.1	23.8 ± 2.2	ns
VFT performance	8.5 ± 2.5	9.5 ± 3.3	ns
MMSE	29.0 ± 1.1	28.9 ± 1.2	ns*^b^*
HAMA	8.7 ± 3.4	4.7 ± 3.1	**0.006**
HAMD	6.2 ± 3.2	4.1 ± 2.8	ns
**Executive functions**			
Clock Drawing Test	9.3 ± 0.8	8.9 ± 1.0	ns*^b^*
Semantic Fluency Test (animals)	22.2 ± 8.9	19.9 ± 5.0	ns
**Language**			
30-item Boston Naming Test	26.0 ± 2.3	25.7 ± 2.1	ns
Similarities Test	19.8 ± 3.7	18.6 ± 3.8	ns
**Attention/working memory**			
Symbol Digit Test	52.2 ± 18.6	36.7 ± 10.2	**0.032** ^^b^^
Digit Span Test (total)	16.1 ± 2.3	14.5 ± 2.1	**0.058**
Forward digit span	9.8 ± 1.4	9.2 ± 1.3	ns*^b^*
Backward digit span	6.3 ± 1.3	5.1 ± 1.2	**0.035** *^b^*
**Episodic memory**			
Logical memory	13.0 ± 4.9	11.8 ± 3.6	ns
Auditory Verbal Learning Test	41.5 ± 12.0	36.0 ± 6.0	ns
**Visuospatial functions**			
Clock copying (Royall’s CLOX 2)	15.2 ± 0.9	14.8 ± 1.0	ns*^b^*
Block Design Test	30.7 ± 7.0	31.6 ± 6.4	ns

^a^χ^2^ test; ^b^ Mann-Whitney U test; ns, non-significant.

### Brain activation during VFT within two groups

**FIGURE 2:**
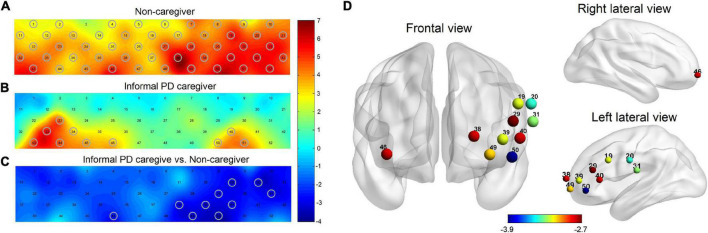
Brain activation maps of oxy-Hb level in non-caregiver and informal PD caregiver during VFT. **(A)** Brain activation during VFT for non-caregiver; **(B)** brain activation during VFT for informal PD caregiver; **(C)** the group comparison for brain activation between the informal PD caregiver and non-caregiver; red/yellow/green represents positive activation and blue represents negative activation in the **(A−C)**. **(D)** The locations of the corresponding brain regions of the significant channels in **(C)**, red to blue represents the increasing degree of negative activation. Gray cycles are utilized to label channels that achieve statistical significance. All *p* < 0.05, FDR corrected.

All 52 channels showed a significant increase in oxy-Hb changes during the task, except for Ch 3, in non-caregivers (*p* = 0.029, FDR corrected) (Figure 2A). In the PD-caregiver group, the oxy-Hb changes were significantly enhanced in Ch23, 33, 34, 40, 43–46, 50, and 51 (*p* = 0.005, FDR corrected) (Figure 2B). Gray cycles were used to label channels that achieved statistical significance.

### Brain activation during verbal fluency task between two groups

PD caregivers exhibited significantly lower cortical activation than the non-caregiver group. Markedly lower increases in oxy-Hb were detected on 10 channels in the PD-caregiver group, (Ch19, 20, 29, 31, 38–40, 46, 49, and 50) with FDR corrected *p* = 0.012, compared to the non-caregiver group. In Figure 2C, gray circles mark the channels that achieve statistical significance between the two groups. These 10 channels correspond to the following brain areas: the left DLPFC (Ch 38 and 39), the left VLPFC (Ch 19, 29, 40, and 50), the bilateral OFC (Ch 46 and 49), and the bilateral SMA (Ch 20 and 31) (Figure 2D).

### Resting-state functional connectivity within and between two groups

**FIGURE 3:**
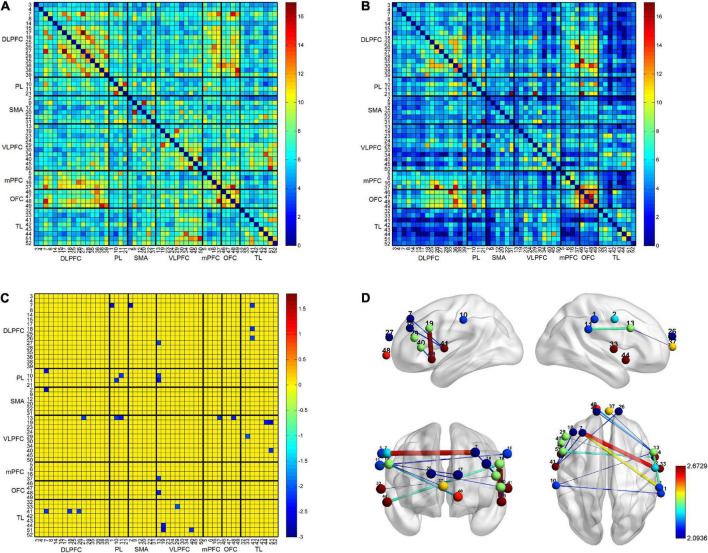
The results of the RSFC pattern within and between non-caregiver and informal PD caregiver. The RSFC within and between the bilateral frontal, parietal, and temporal lobes was markedly increased in the non-caregiver group **(A)** (*p* < 0.05, FDR corrected) and showed a lower increase in the informal PD-caregiver group **(B)** (*p* < 0.05, FDR corrected). Group comparison showed the RSFC decreased in informal PD caregiver within the bilateral IPL and between the left DLPFC and the right IPL, the right SMA, the right VLPFC, and the left TL, between the left VLPFC and the bilateral TL, between the right VLPFC and the left OFC, the mPFC, and the bilateral IPL, and between the right DLPFC and the left TL **(C,D)** (*p* < 0.05, uncorrected).

The results of the RSFC pattern and the *t*-test for the PD-caregiver and non-caregiver groups are presented in Figure 3. A one-sample *t*-test revealed that there was a significant increase in the RSFC within and between the bilateral frontal, parietal, and temporal lobes in the non-caregiver group (*p* = 0.014, FDR corrected) (Figure 3A) and a lower increase in the PD-caregiver group (*p* = 0.047, FDR corrected) (Figure 3B). In the PD-caregiver group, the RSFC decreased within the bilateral IPL and between the left DLPFC and the right IPL, the right SMA, the right VLPFC, and the left TL, between the left VLPFC and the bilateral TL, between the right VLPFC and the left OFC, the mPFC, and the bilateral IPL, and between the right DLPFC and left TL (*p* = 0.014 ∼ 0.048, uncorrected) compared with the non-caregiver group (Figures 3C,D), which indicated hypo-connectivity of the brain network within FPN as well as the decoupling of the DMN with the FPN.

### Correlation between verbal fluency task-induced activation and clinical variables

**FIGURE 4:**
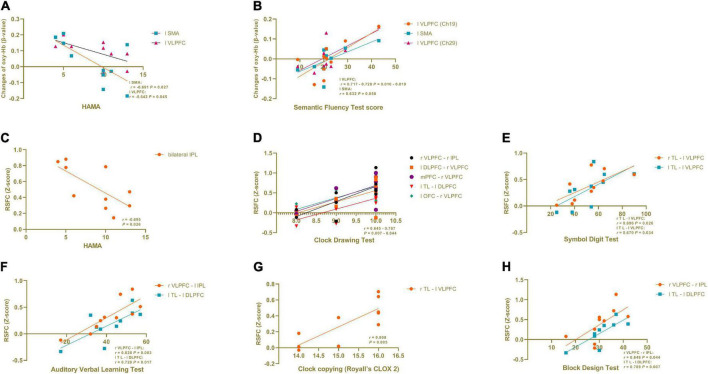
Correlation analysis in informal PD caregiver between clinical variables and brain activation during VFT and in RSFC. **(A)** The HAMA score showed a significant negative correlation with the oxy-Hb level (β-value) in the left SMA and the left VLPFC during VFT. **(B)** The Semantic Fluency Test score was positively correlated with the oxy-Hb level in the left VLPFC and the left SMA during VFT. **(C)** The HAMA score showed a significant negative correlation with the RSFC within the bilateral IPL. **(D−H)** Cognitive domain tests showed a positive correlation with the RSFC between different brain regions. All *p* < 0.05.

In the PD-caregiver group, the HAMA score showed significant negative correlation with the oxy-Hb level (β-value) in the left SMA (Ch 31) (*r* = −0.691, *p* = 0.027) and the left VLPFC (Ch 40) (*r* = −0.642, *p* = 0.045). The Semantic Fluency Test score was positively correlated with the oxy-Hb level in the left VLPFC and the left SMA (Ch 19, 20, and 29) (*r* = 0.632 – 0.728, *p* = 0.016 – 0.050). The results are shown in Figure 4. No significant correlation was observed between the oxy-Hb level changes and other clinical variables such as the HAMD, the MMSE, VFT performance, and the Clock Drawing Test.

### Correlation analysis between resting-state functional connectivity and clinical variables

As seen in Figure 4, in the PD-caregiver group, the HAMA score showed a significant negative correlation with the RSFC within the bilateral IPL (*r* = −0.695, *p* = 0.026). The Clock Drawing Test was positively correlated with the RSFC between the right VLPFC and the right IPL, the left DLPFC and the right VLPFC, the mPFC and the right VLPFC, the left TL and the left DLPFC, and the left OFC and the right VLPFC (*r* = 0.645 – 0.787, *p* = 0.007 – 0.044). The Symbol Digit Test was positively correlated with the RSFC between the right TL and the left VLPFC (*r* = 0.696, *p* = 0.026) and between the left TL and the left VLPFC (*r* = 0.670, *p* = 0.034). The Auditory Verbal Learning Test was positively correlated with the RSFC between the right VLPFC and the left IPL (*r* = 0.828, *p* = 0.003) and between the left TL and the left DLPFC (*r* = 0.728, *p* = 0.017). The Clock copying (Royall’s CLOX 2) was positively correlated with RSFC between the right TL and the left VLPFC (*r* = 0.808, *p* = 0.005). The Block Design Test was positively correlated with the RSFC between the right VLPFC and the right IPL (*r* = 0.64 *p* = 0.044) and between the left TL and the left DLPFC (*r* = 0.789 *p* = 0.007). Collectively, the above findings indicate that cognitive function was positively associated with the RSFC within the FPN and between the DMN and the FPN in the PD-caregiver group.

## Discussion

In the present study, we evaluated comprehensive clinical scale data and explored the neural mechanisms of executive tasks and the RSFC in neural networks underlying informal PD caregivers. Several noteworthy findings were observed. First, informal PD caregivers experienced significantly higher levels of anxiety than non-caregivers. Second, we found that informal PD caregivers showed no differences in most cognitive domain functions compared to non-caregivers while performing better in the attention functions. Third, decreased task-evoked activity in the left DLPFC, the left VLPFC, the bilateral OFC, and the bilateral SMA was observed during VFT in informal PD caregivers. This was accompanied by reduced RSFC within FPN and decoupling of the FPN with the DMN. Fourth, in the PD caregiver, task-driven activity in the VLPFC and the SMA and the RSFC within the bilateral IPL were negatively correlated with the HAMA. Finally, the RSFC within the FPN and between the DMN and the FPN was positively associated with cognitive domain functions in informal PD caregivers. These results support the hypothesis that dysfunction of the prefrontal cortex (PFC) and the RSFC in the neural network are present in informal PD caregivers, and the dysfunctions are associated with anxiety levels.

Compared to non-caregivers, informal PD caregivers showed more anxiety symptoms, as supported by a previous study (9, 52). Several reasons may explain the anxiety symptoms. On the one hand, caregivers may lack enough knowledge and skills to properly respond to behavioral and lifestyle problems of the patients (53), while these persistent problems of the patients could further negatively influence the caregivers’ anxiety symptoms. On the other hand, since PD is a progressive neurological condition, the mental and physical needs of the patients increase significantly as the severity of the disease increases, indicating that caregivers have to spend more time and energy caring for the patient (54). The increased care demands undertaken by the caregivers lead to feeling overwhelmed and stressed (1), which ultimately results in anxiety. In addition, caregiver anxiety could, in turn, negatively impact the patient’s health-related quality of life (55). Thus, informal PD caregiver’s mental health is an important concept not only because of its leading role in moderating a caregiver’s burden but also because a caregiver’s health-related quality of life is an isolated contributor to the caregiver’s stress levels (56).

The cognitive performance of informal PD caregivers did not differ from controls in executive, memory, visuospatial, and language functions, but the performance in the attention domain was significantly better. Our findings contrast with most previous studies on informal caregiving and cognition, where caregiving had negative impacts on cognitive functions (57). For example, informal caregiving is related to a decline in cognitive function (57). This could be attributed to the fact that the effect of anxiety on cognition depends on the severity of the current anxiety symptoms (58). In our study, mild anxiety symptoms in informal PD caregivers were observed. Previous studies used cognitive scales to investigate the relationship between anxiety and cognition in elders, suggesting that mild anxiety was related to better cognitive performance, including attention (58, 59). In addition, the healthy caregiver hypothesis argues that caregiving-related factors, such as cognitive care requirements, may help protect caregivers from further strain and contribute to maintaining cognitive health in later life (60). Accordingly, our observation of mild anxiety in caregivers may reflect a compensatory mechanism for cognitive function.

While the cognitive rating scale found better cognitive performance in informal PD caregivers, fNIRS revealed the opposite result, with dysfunction in the prefrontal lobes during the executive task. This finding is particularly interesting. The informal PD group exhibited less activation in the left DLPFC, the left VLPFC, the bilateral OFC, and the bilateral SMA relative to controls during VFT. The DLPFC, a key brain area involved in broadly-defined executive function, is associated with cognitive flexibility, decision making, inhibition, working memory, and response selection (61). The VLPFC and the OFC have direct connections with the DLPFC and are involved in executive function, such as behavioral inhibition, reward representation, decision making, and mnemonic process to maintain and retrieve information in the working memory (19, 61, 62). As key brain regions of the FPN, the SMA are commonly involved in the activation of response inhibition (63). Together, these brain areas were recruited to involve in the executive control process. As such, potential neural substrates of this executive function deficit were observed, with informal PD caregivers showing less recruitment of the frontal and parietal control areas compared with controls during VFT.

Considering inconsistent results between normal cognitive performance and reduced brain activation during the cognitive task in informal PD caregivers, an explanation of the underlying mechanism is required. In our study, significant anxiety symptom was found in informal the PD caregiver than the non-caregiver, while other demographic and clinical assessments did not differ. The majority of neuroimaging studies on anxiety have found a negative effect of anxiety on PFC. A recent systematic review (64), for example, suggested that anxiety could directly disrupt the PFC, affecting the processing of working memory and cognitive executive functions. In addition, a previous study showed that PFC recruitment during the performing of executive function was reduced in an individual with non-clinical anxiety (65), consistent with cognitive dysregulation in anxiety disorder (66). Therefore, the possible explanation for these results could be that mild (non-clinical) anxiety symptoms in informal PD caregivers affect the cortical resources during executive function but that anxiety does not impair performance in neuropsychological assessment.

In this study, the decreased RSFC between the DLPFC and the TL, the VLPFC and the TL, the VLPFC and the mPFC, and the VLPFC and the OFC, which may reflect the impaired connectivity between the DMN and the FPN, was weaker in informal PD caregivers than in controls. In addition, the decreased RSFC within the bilateral IPL and between the DLPFC and the IPL, the DLPFC and the VLPFC, and the VLPFC and the IPL may reflect the reduced connectivity in FPN. Together, our findings show that informal PD caregiver is featured with hypo-connectivity of the brain network within FPN as well as the decoupling of the FPN with the DMN. The DMN and the FPN are commonly shown to increase their activity in multiple forms of complex or higher-level cognition, such as memory, abstract thinking, versatile reasoning, and cognitive control (21–23). We believe the specific changes to neural functional networks in informal PD caregivers, both intra-networks and inter-networks, are implicated in anxiety symptoms (67), similar reasons to task-based results. In anxious individuals, reduced RSFC of the DMN and the FPN is likely to be associated with adverse cognitive emotion regulation, which is considered a core characteristic of the neurophysiology of anxiety (66, 68, 69). Decreased RSFC between the DMN and the FPN may reflect and exhibit diminished executive control (67), and this abnormal control may be reflected as worrying conditions (70). Thus, our results suggest that hypo-connectivity between the DMN and the FPN and within the FPN may be a characteristic feature of the caregivers, which is affected by anxiety.

Interestingly, during VFT, a negative correlation was found in informal PD caregivers between anxiety severity and activation in the VLPFC and the SMA, while a positive correlation was observed between the Semantic Fluency Test score and the activation in the VLPFC and the SMA. The VLPFC and the SMA, as key brain regions for FPN, are involved in executive functions (18, 71). Our findings support previous reports that anxiety severity is inversely associated with prefrontal activation (66), and executive performance was positively correlated with prefrontal activation (72). Such findings indicate that failing to engage the PFC during executive tasks (VFT) may be related to the anxiety symptoms of informal PD caregivers.

Similarly, altered intrinsic connectivity between or within networks was observed in resting state. The RSFC within the FPN was inversely correlated with the severity of anxiety in line with the resting-state studies that indicate that the RSFC of the FPN was negatively correlated with the anxiety level (73, 74), indicating that dysfunction in the FPN may be associated with anxiety in informal PD caregivers. Furthermore, the RSFC between the DMN and the FPN and within FPN was positively correlated with cognitive function, including execution, attention/working memory, memory, and visuospatial function. This observation may be in line with the fact that the DMN and the FPN increased their activity in multiple forms of cognitive process and were positively associated with cognitive performance (75). Interestingly, the correlation analyses of the RSFC exhibited relatively consistent alterations observed in the task-based data and indicated the severity of anxiety-influenced neural activation of the FPN as well as coupling of the FPN with the DMN.

This research has several limitations that should be addressed in future investigations. First, since this is a preliminary study with a comparatively small sample size, the findings should be confirmed in a larger population. Second, this study did not include a detailed description of the PD caregiver burdens, such as physical burden, psychological burden, social burden, financial burden, and comprehensive clinical features of the care recipient (10, 14). In future studies, the inclusion of detailed information on informal PD caregivers using a scale of caregiver burden [e.g., the Caregiving Burden Scale (76)] may help to further explain the results of the study. Third, the cognitive modulation mechanisms in informal PD caregivers are still not fully understood as the 52-channel ETG-4000 device does not cover the whole brain. Future studies should adopt the fNIRS device covering more brain regions to fully explore the neural basis of cognitive dysregulation in informal caregivers with PD. Finally, the spatial resolution of fNIRS is lower than that of fMRI. However, most of our findings are consistent with previous studies of anxiety individuals observed by other neuroimaging modalities.

## Conclusion

To our knowledge, this is the first study on informal PD caregivers performing comprehensive cognitive and clinical assessment and exploring neural activity related to the executive task and brain connectivity during the resting state by fNIRS. In the study, we confirmed that informal PD caregivers had no difference in most cognitive domain functions but had better attentional functions, along with higher anxiety levels, compared to non-caregivers. Besides, compared with controls, informal PD caregivers showed lower activation over the prefrontal control regions during the executive task and hypo-connectivity within FPN and between DMN and FPN in the resting state, which are attributed to the negative effect of anxiety on the brain. Such findings could increase our understanding of the functional brain deficits of informal PD caregivers and emphasize the importance of addressing the mental health issues of informal caregivers assisting the PD patients.

## Data availability statement

The original contributions presented in this study are included in the article/supplementary material, further inquiries can be directed to the corresponding authors.

## Ethics statement

The studies involving human participants were reviewed and approved by The Ethical Committee of the First Affiliate Hospital of Dalian Medical University. The patients/participants provided their written informed consent to participate in this study.

## Author contributions

H-YW, CS, and ZL designed the study. H-YW analyzed the fNIRS data. LR, TL, LP, and XH were in charge of collecting the fNIRS data and assisted in the fNIRS data analysis. H-YW, SW, CS, and ZL recruited the subjects. H-YW, LR, CS, and ZL collected the demographic, clinical, and neuropsychological information of the subjects. H-YW wrote the manuscript. All authors have read and approved the final manuscript.
